# Standardization of perioperative care facilitates safe discharge by postoperative day five after pancreaticoduodenectomy

**DOI:** 10.1371/journal.pone.0209608

**Published:** 2018-12-28

**Authors:** Sara K. Daniel, Lucas W. Thornblade, Gary N. Mann, James O. Park, Venu G. Pillarisetty

**Affiliations:** University of Washington Department of Surgery, Seattle, WA, United States of America; University of Florida, UNITED STATES

## Abstract

**Introduction:**

Pancreaticoduodenectomy is a complex surgical procedure associated with high morbidity and prolonged length of stay. Enhanced recovery after surgery principles have reduced complications rate and length of stay for multiple types of operations. We hypothesized that implementation of a standardized perioperative care pathway would facilitate safe discharge by five days after pancreaticoduodenectomy.

**Methods:**

We performed a retrospective cohort study of patients undergoing pancreaticoduodenectomy 18 months prior to and 18 months following implementation of a perioperative care pathway at a quaternary center performing high volume pancreatic surgery.

**Results:**

A total of 145 patients underwent pancreaticoduodenectomy (mean age 63 ± 10 years, 52% female), 81 before and 64 following pathway implementation, and the groups were similar in terms of preoperative comorbidities. The percentage of patients discharged within 5 days of surgery increased from 36% to 64% following pathway implementation (p = 0.001), with no observed differences in post-operative serious adverse events (p = 0.34), pancreatic fistula grade B or C (p = 0.28 and p = 0.27 respectively), or delayed gastric emptying (p = 0.46). Multivariate regression analysis showed length of stay ≤5 days three times more likely after pathway implementation. Rates of readmission within 30 days (20% pre- vs. 22% post-pathway (p = 0.75)) and 90 days (27% pre- vs. 36% post-pathway (p = 0.27)) were unchanged after pathway implementation, and were no different between patients discharged before or after day 5 at both 30 days (19% ≤5 days vs. 23% ≥ 6 days (p = 0.68)) and 90 days (32% ≤5 days vs. 30% ≥ 6 days (p = 0.81)).

**Conclusions:**

Standardizing perioperative care via enhanced recovery protocols for patients undergoing pancreaticoduodenectomy facilitates safe discharge by post-operative day five.

## Introduction

Pancreaticoduodenectomy (PD) is a complex operation associated with morbidity rates approaching 65%[1–3]. Patients are typically hospitalized for over one week, and often require a stay in the intensive care unit (ICU)[4,5]. Factors which may have contributed to this long length of stay (LoS) include historical emphasis on pancreatic and nasogastric drainage, as well as prolonged post-operative bowel rest[6–10]. With the implementation of enhanced recovery after surgery (ERAS) or "fast track" post-operative pathways in the 1990s, these traditional tenets of post-operative surgical recovery have been challenged[11]. While a majority of ERAS literature has focused on colorectal surgery, recent attempts have been made to translate these principles to other sub-specialties, including hepatopancreatobiliary surgery[12]. Generalized concepts in ERAS pathways focus on multimodal narcotic-sparing pain control, early ambulation, and advancement of diet, and require active participation from patients and providers at all levels[7–10,13–15].

In aiming for timely discharge for our patients, we determined that there were opportunities for refinement of our perioperative care practices. We subsequently convened a multi-disciplinary care team comprised of clinic, operating room, and ward staff to develop a standardized peri-operative PD pathway. In addition to the nurses, advanced practice providers, and physicians who provide care in these settings, stakeholders from physical and occupational therapy, social work, nutrition, and pharmacy were actively involved in discussions. This multidisciplinary team examined the highest available level of evidence to create the pathway that was ultimately implemented by all practitioners[15].

**Fig 1:**
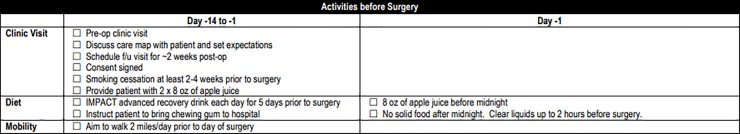
Whipple Pre-operative Pathway.

**Fig 2:**
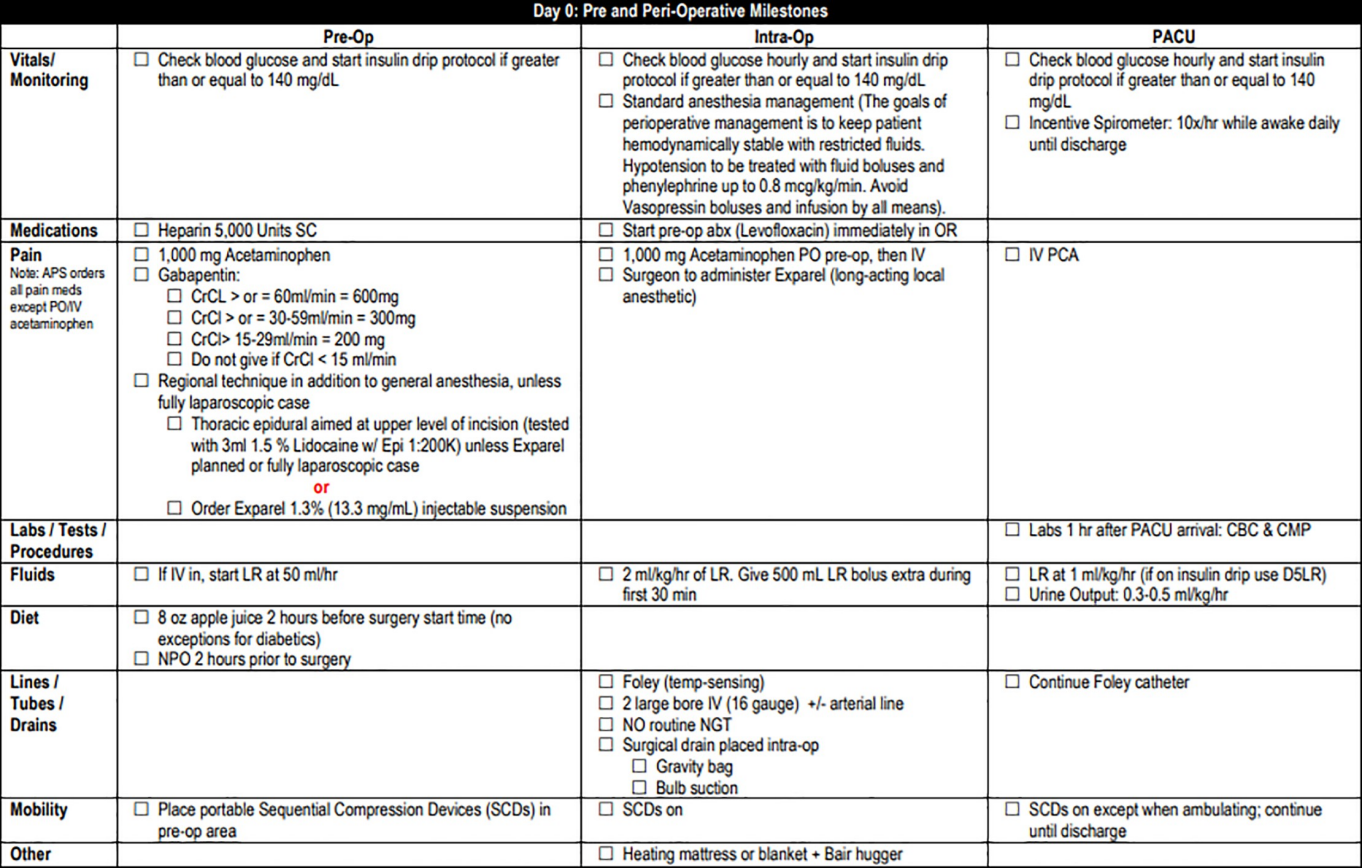
Whipple Intra-operative Pathway.

**Fig 3:**
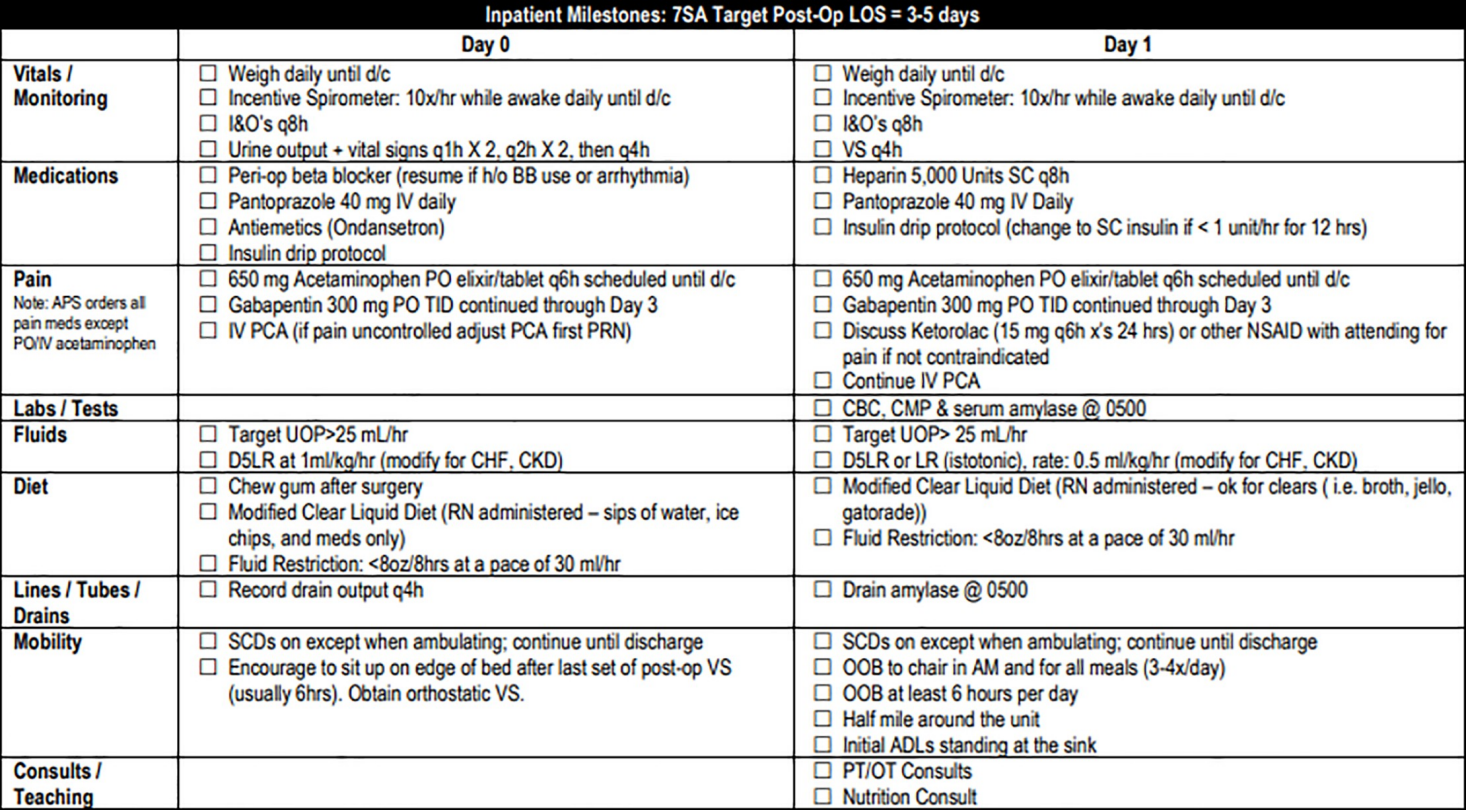
Whipple Post-operative Pathway Days 0–1.

**Fig 4:**
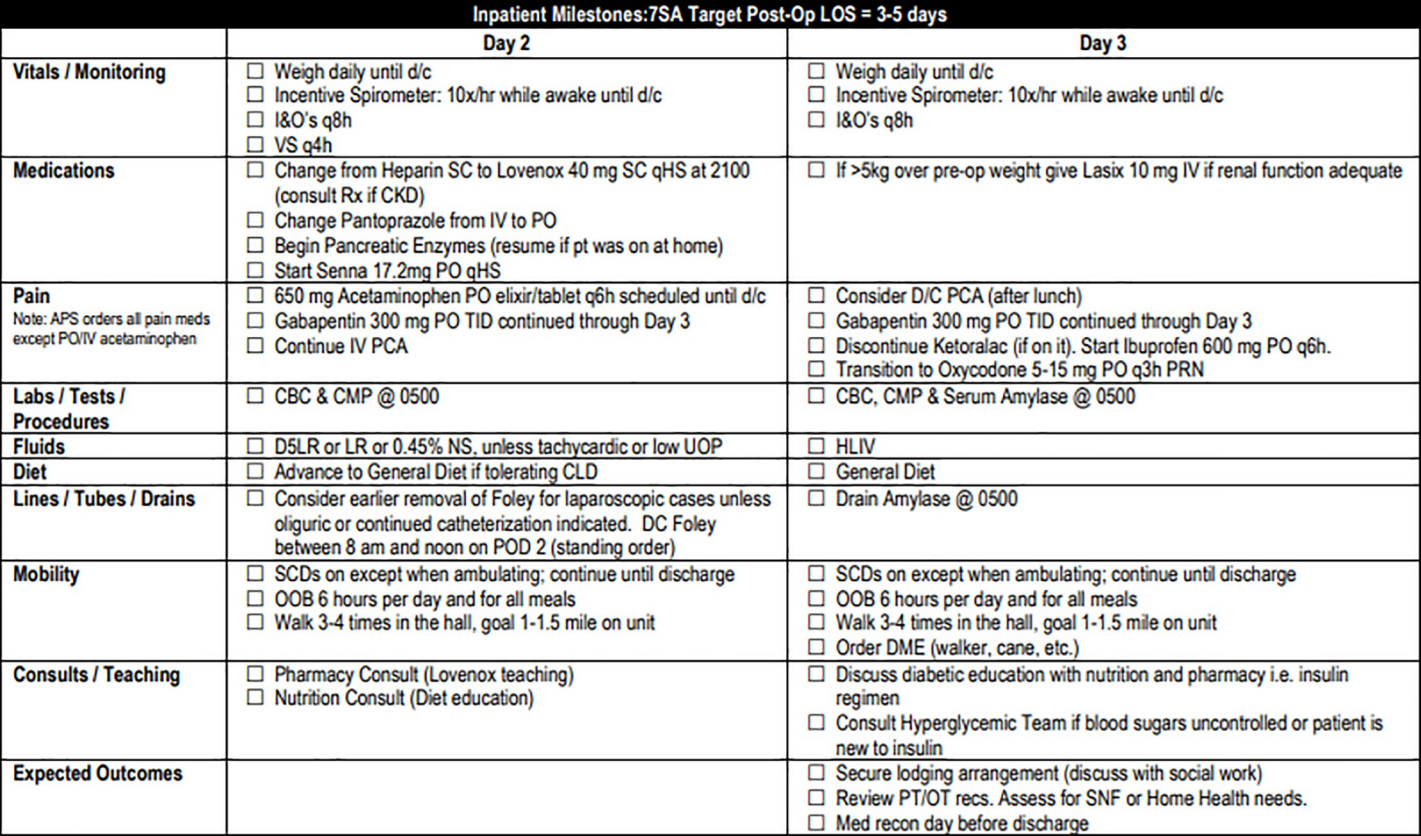
Whipple Post-operative Pathway Days 2–3.

**Fig 5:**
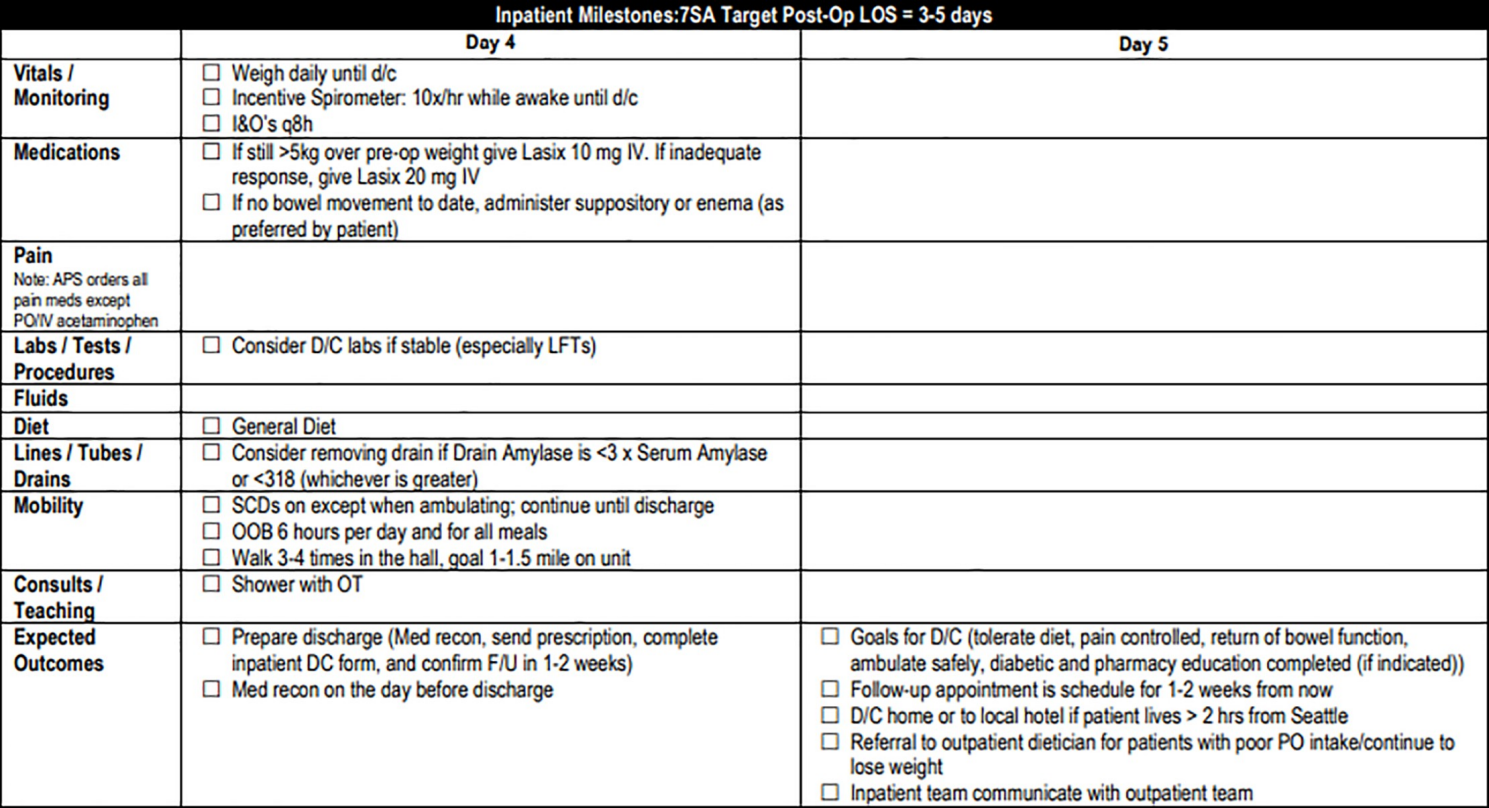
Whipple Post-operative Pathway Days 4–5.

Pre-operative components of the pathway included teaching by clinic nurses emphasizing expected post-operative activity level, which is higher than many patients originally anticipate. Other topics included the gradual return to pre-surgery activity level at home, as well as the importance of adequate nutrition for the healing process. Our clinic staff reviews handouts in person with the patient and family during pre-operative teaching (S1 Fig). Additionally, patients are encouraged to increase their protein intake prior to surgery including arginine rich nutrition shakes 5 days before surgery. (Fig 1). In order to reduce insulin resistance, patients received oral carbohydrate loading two hours prior to surgery. Intra-operative blood glucose levels were monitored and maintained at or below a target of 140 mg/dL[15,16]. Intravenous (IV) fluids in the OR were targeted to a goal of 2mL/kg/hr with an extra 500mL bolus in the first 30 minutes based loosely on the RELIEF protocol[17]. Nasogastric (NG) tubes were not routinely placed intra-operatively (Fig 2). Patients were started on clear liquids on post-operative day (POD) 1 and advanced to regular diet on POD 2 or 3, as tolerated (Fig 3). Criteria used to determine diet advancement included lack of nausea, bloating, or excessive belching, however, this was ultimately a patient-specific clinical decision. IV fluids were targeted at 1mL/kg/hr on POD 0 and 0.5 mL/kg/hr on POD 1 for most patients in balance with a target urine output of at least 25mL/hr. Goal was discontinuation of IV fluids in conjunction with diet advancement on POD 3. Early and frequent mobility is promoted by having physical and occupational therapy staff evaluate patients on POD 1. Patients are assisted to sit at the edge of the bed on the day of surgery and are OOB walking on POD 1 onwards. (Fig 4). Prophylactic anticoagulation with subcutaneous heparin is instituted preoperatively on the day of surgery and transitioned to daily enoxaparin on POD 3 for a total four-week course[18,19]. All patients had a drain placed intraoperatively in the resection bed with drain and serum amylase levels being checked on POD 1 and 3 to facilitate early drain removal when appropriate. All patients are cleared by nutrition, physical therapy, and occupational therapy prior to discharge with an approximate target of POD 5 (Fig 5). Prior to discharge, a post-operative follow up visit was scheduled for 4 to 14 days after discharge, and patients were provided with clinic and emergency after hours contact information. It is institution policy that all patients receive a discharge follow up phone call 24 to 48 hours after leaving the hospital by a nurse manager who can communicate concerns with the surgical team.

We hypothesized that routine discharge on POD 5 after pancreaticoduodenectomy would be feasible and safe, and that the percentage of patients who were able to reach this milestone would increase after perioperative pathway initiation. We also aimed to examine if there were any particular perioperative variables that were associated with failure to meet goal discharge.

## Methods

We performed a retrospective review of consecutive patients undergoing PD between December 1st, 2013 and November 30th, 2016. After obtaining Institutional Review Board approval of our quality improvement project, a single physician performed the chart review of all PDs identified by CPT code. All procedures were performed by one of three surgeons at our institution, all with over 5 years of hepatobiliary practice. The single pylorus-preserving operation performed during the study period was not included due to the rarity of this procedure at our institution. The remainder of operations were classic open PDs with one surgeon performing some of the initial dissection laparoscopically with planned open completion. Patients were categorized into two treatment groups: before pathway implementation (December 1st, 2013 to May 31st, 2015) and after pathway implementation (June 1st, 2015 to November 30th, 2016). Patient demographics, comorbidities, and outcomes including complications were abstracted using National Surgical Quality Improvement Program (NSQIP) templates[20].

Pancreatic duct leak and delayed gastric emptying (DGE) was defined by the ISGPS 2016 and 2007 criteria respectively[21,22]. Serious adverse events were defined as any post-operative complication tracked by NSQIP other than superficial surgical site infection including cardiac, respiratory, and renal dysfunction as well as infection.

Univariate analysis was conducted with Student's t-test, Kruskal Willis H-test, and chi-square test as appropriate for normally-distributed continuous, non-normally distributed continuous, and categorical variables, respectively. In order to control for differences between patients pre- and post-pathway implementation, we applied multivariate analysis. We selected, a priori, variables thought clinically to be associated with hospital LoS. We also included variables that were significantly associated on univariate analysis with the outcome of LoS ≤5 days. We performed regression analysis to estimate the association of the PD pathway with LoS ≤5 days. We explored nested models using likelihood ratio testing. Analyses were performed with IBM SPSS Statistics 22 and Stata IC v14.0 (StataCorp, College Station, Tx).

## Results

A total of 145 patients underwent PD during the course of this study, 81 pre- and 64 post-pathway implementation. Complete 30-day follow up information was available for 139 (96%) patients, and 90 day follow up was available for 128 (88%) with the others being lost to follow up. Average age was similar pre- and post-pathway implementation (63.8 vs. 63.5 years, p = 0.35) (Table 1). The majority of operations were performed for pancreatic adenocarcinoma in both groups (61% vs. 65%, p = 0.33) and there was no difference in percentage needing vascular resection (28% vs. 34%, p = 0.44). The groups were largely similar in the rate of preoperative comorbidities including COPD (9% vs. 3%, p = 0.18) and diabetes (21% vs. 20%, p = 0.92), however there was a higher rate of insulin use in the pre-pathway group (p = 0.04). There was no significant difference in CKD between groups as defined by GFR <60 ml/min/1.73m^2^ (6% vs. 0%, p = 0.29), but there were more cardiac stents placed in the post-pathway group (2% vs. 13%, p = 0.02). Comorbidity and functional scoring via American Society of Anesthesiologists physical status classification (ASA) (22% vs. 29% ≤2,p = 0.37) and Eastern Cooperative Oncology Group (ECOG) performance status (82% vs 91% ≤1, p = 0.06) were not significantly different between the two groups. Pre-operative albumin levels < 3.5 g/dL were not different pre- and post-pathway implementation (34% vs 29%, p = 0.61). There was no significant difference in the proportion of patients who had neoadjuvant chemotherapy pre- and post-pathway implementation (26% vs. 38%, p = 0.14), but there was an increase in the use of neoadjuvant radiation post-pathway (14% vs. 28%, p = 0.03). Percentage of patients requiring vascular resections was not different before and after pathway implementation (28% vs. 34%, p = 0.44). Epidurals (44% vs. 8%, p<0.001) and NG tubes (22% vs. 8%, p = 0.02) were placed intra-operatively more commonly before pathway implementation.

**Table 1:** Demographics, clinical characteristics, and outcomes of patients before and after PD pathway implementation.

	Before Pathway (n = 81)	After Pathway (n = 64)	All patients (n = 145)	p-value
**Demographics**				
Mean age, years (SD)	63.3 (11.2)	63.5 (9.6)	63.4 (10.5)	0.35
Female, n (%)	40 (49)	36 (56)	76 (52)	0.41
**Clinical characteristics**				
Mean BMI, kg/m2 (SD)	26.9 (5.9)	25.8 (4.8)	26.4 (5.4)	0.33
Mean smoking pack years (SD)	10.9 (21.3)	9.2 (18.7)	10.1 (20.1)	0.78
Diabetes, n (%)	17 (21)	13 (20)	30 (20)	0.92
Previous cardiac stent (%)	2 (2)	8 (13)	10 (7)	**0.02**
CKD, n (%)	4 (6)	0 (0)	4 (4)	0.29
Hypoalbuminemia <3.5 g/dL, n (%)	20 (34)	10 (29)	30 (32)	0.61
ECOG performance status, n (%)				0.06
0	26 (55)	22 (40)	48 (47)	
1–2	19 (40)	33 (60)	52 (51)	
≥3	2 (4)	0 (0)	2 (2)	
ASA class, n (%)				0.370
1	1 (1)	0 (0)	1(1)	
2	17 (21)	19 (30)	36 (25)	
≥3	63 (78)	45 (70)	108 (74)	
Neoadjuvant chemotherapy, n (%)	21 (26)	24 (38)	45 (31)	0.14
Neoadjuvant radiation, n (%)	11 (14)	18 (28)	29 (20)	**0.03**
Pre-operative epidural, n (%)	36 (44)	5 (8)	41 (28)	**<0.001**
Mean IV fluid in OR, mL (SD)	5,710 (1,971)	4,866 (2,297)	5,337 (2,155)	**0.01**
Blood transfusion in OR, n (%)	7 (8)	11 (17)	18 (12)	0.12
Mean EBL in OR, mL (SD)	451.7 (860.6)	451.3 (622.8)	451.6 (762.4)	0.99
Soft pancreatic texture, n (%)	31 (45)	22 (36)	53 (41)	0.30
Pancreatic duct <3mm, n (%)	24 (33)	22 (35)	46 (34)	0.79
**Primary outcome**				
LOS ≤5 days, n (%)	29 (36)	41 (64)	70 (48)	**0.001**
**Secondary outcomes**				
ICU admission POD1, n (%)	10 (12)	9 (14)	19 (13)	0.81
Serious adverse event, n (%)	22 (27)	22 (34)	44 (30)	0.34
ISGPS 2016 grade B leak, n (%)	19 (23)	10 (16)	29 (20)	0.28
ISGPS 2016 grade C leak, n (%)	5 (6)	7 (11)	12 (8)	0.27
Delayed gastric emptying grade B or C, n (%)	17 (21)	10 (16)	27 (18)	0.46
Required TPN post-operatively, n (%)	16 (19)	8 (12)	24 (16)	0.27
Discharge to other place than home, n (%)	6 (7.4)	2 (3.1)	8 (5.5)	0.26
30 day readmission, n (%)	16 (20)	14 (22)	30 (21)	0.75
90 day readmission, n (%)	19 (27)	21 (36)	40 (31)	0.27

PD, Pancreaticoduodenectomy; SD, Standard deviation; BMI, Body mass index; CKD, Chronic kidney disease; ECOG, Eastern Cooperative Oncology Group performance status; ASA, American Society of Anesthesiologists physical status classification; LOS, Length of stay; ISGPS, International Study Group of Pancreatic Surgery; IV, Intravenous; OR, Operating room; EBL, Estimated blood loss

The proportion of patients discharging by a target of ≤5 days was nearly two times higher post-pathway implementation (36% vs. 64%, p = 0.001) for a median LoS of 5 days post pathway compared to 6 days pre-pathway (Table 2). Serious adverse events (SAEs) were similar between both the pre- and post-pathway groups (27% vs. 34%, p = 0.34). Rate of pancreatic leak grade B or C by ISGPS definition (29% vs. 27% p = 0.35) did not reach statistical significance^21^. Discharge to location other than home was not significantly different post after pathway implementation (7% vs. 3%, p = 0.26). Readmission within 30 days(20% pre- vs. 22% post-pathway (p = 0.75)) and 90 days (27% pre- vs. 36% post-pathway (p = 0.27)) were not significantly different, nor was the number of days from discharge to readmission was significantly higher post-pathway implementation (day 30.4 vs. day 29.8, p = 0.95). Needing to be placed on total parenteral nutrition (TPN) (19% vs. 12%, p = 0.27) or having an NG tube placed in the post-operative period (after removal of the intra-operative NG if placed) (19.1% vs. 17.1%, p = 0.69) were not different between pre- or post-pathway groups. Rates of grade B or C DGE by ISGPS criteria (21% vs. 16%, p = 0.46) were not different pre- to post-pathway[22]. Of note, 5 patients pre-pathway and 2 patients post pathway included in the DGE cohort were tolerating a solid diet, but were made NPO and started on TPN due to a persistent pancreatic leak. Looking only at patients without any post-operative SAEs, 24 of 59 pre-pathway patients (41%) were discharged in 5 days or less for a median LoS of 6 days (mean 6.5). Post-pathway, 22 of 42 patients without post-operative SAEs (52%) were discharged by goal of 5 days or less for a median LoS of 5 days (mean 5.0). The difference in reaching goal LoS between the pre-and post-pathway groups remain significant with p<0.001 without increase in readmission within 30 days of surgery (p = 0.93).

**Table 2:** Demographics, clinical characteristics, and outcomes of patients with LOS within goal of 5 days after PD or not.

	LoS ≤ 5 days (n = 70)	LoS ≥ 6 days (n = 75)	All patients (n = 145)	p-value
**Demographics**				
Mean age, years (SD)	64.0 (10.4)	62.8 (10.6)	63.4 (10.5)	0.16
Female, n (%)	36 (51)	40 (53)	76 (52)	0.81
**Clinical characteristics**				
Mean BMI, kg/m2 (SD)	25.6 (4.6)	27.2 (6.0)	26.4 (5.4)	0.13
Mean smoking pack years (SD)	6.5 (11.3)	13.5 (25.4)	10.1 (20.1)	0.20
Diabetes, n (%)	14 (20)	16 (21)	30 (20)	0.84
Previous cardiac stent (%)	7 (10)	3 (4)	10 (7)	0.19
CKD, n (%)	1 (2)	3 (5)	4 (4)	0.62
Hypoalbuminemia <3.5 g/dL, n (%)	8 (19)	22 (44)	30 (32)	**0.01**
ECOG performance status, n (%)				0.36
0	26 (51)	22 (43)	48 (47)	
1–2	25 (49)	27 (53)	52 (51)	
≥3	0 (0)	2 (4)	2 (2)	
ASA class, n (%)				0.43
1	1 (1)	0 (0)	1(1)	
2	18 (26)	18 (24)	36 (25)	
≥3	51 (73)	57 (76)	108 (74)	
Neoadjuvant chemotherapy, n (%)	17 (24)	28 (37)	45 (31)	0.09
Neoadjuvant radiation, n (%)	14 (20)	15 (20)	29 (20)	1.00
Preop epidural, n (%)	13 (18)	28 (37)	41 (28)	**0.01**
Mean IV fluid in OR, mL (SD)	4,718 (2,023)	5,915 (2,126)	5,337 (2,155)	**0.001**
Blood transfusion in OR, n (%)	8 (11)	10 (13)	18 (12)	0.72
Mean EBL in OR, mL (SD)	433.3 (932.4)	468.6 (565.4)	451.6 (762.4)	0.78
Soft pancreatic texture, n (%)	25 (37)	28 (45)	53 (41)	0.40
Pancreatic duct <3mm, n (%)	22 (31)	24 (37)	46 (34)	0.46
**Secondary outcomes**				
ICU admission POD1, n (%)	2 (3)	17 (22)	19 (13)	**<0.001**
Serious adverse event, n (%)	14 (20)	30 (40)	44 (30)	**0.009**
ISGPS 2016 grade B leak, n (%)	*9 (12)	20 (27)	29 (20)	**0.03**
ISGPS 2016 grade C leak, n (%)	*3 (4)	9 (12)	12 (8)	0.08
Delayed gastric emptying grade B or C, n (%)	*5 (7)	22 (30)	27 (18)	**<0.001**
Required TPN post-op, n (%)	*4 (5)	20 (27)	24 (16)	**0.001**
Discharge to other place than home, n (%)	0 (0.0)	8 (10.6)	8 (5.5)	**0.005**
30 day readmission, n (%)	13 (19)	17 (23)	30 (21)	0.68
90 day readmission, n (%)	20 (32)	20 (30)	40 (31)	0.81

*All diagnosed during readmission

PD, Pancreaticoduodenectomy; SD, Standard deviation; BMI, Body mass index; CKD, Chronic kidney disease; ECOG, Eastern Cooperative Oncology Group performance status; ASA, American Society of Anesthesiologists physical status classification; LOS, Length of stay; ISGPS, International Study Group of Pancreatic Surgery; IV, Intravenous; OR, Operating room; EBL, Estimated blood loss

The 30-day readmission rates of all patients discharged ≤ 5 days after surgery (n = 70) was similar that of patients discharged ≥ 6 days following surgery (n = 75; 19% vs. 23%; p = 0.68). There remained no significant difference in 90 day readmission after surgery for those discharged in ≤ 5 days or ≥ 6 days (32% vs. 30%, p = 0.81). Furthermore, the average date of readmission was similar between patients with LoS ≤ 5 and ≥ 6 (27.1 vs. 33.6 days; p = 0.53). LoS ≥ 6 days was associated with increased serious adverse events (20% vs. 40%, p = 0.009) and discharge to a location other that home (0% vs. 10%, p = 0.005). Looking at in-hospital risk factors for increased LoS, epidural placement was significantly associated with LoS ≥ 6 days (18% vs. 37%, p = 0.01). Intra-operative NG placement was higher in those with LOS ≥ 6 days (8% vs. 23%, p = 0.018) as was need for TPN (5% vs. 27%, p = 0.001) and grade B or C DGE (7% vs. 30%, p<0.001). Pancreatic leak grade B or C by ISGPS criteria was also associated with LoS ≥ 6 days (17% vs. 39%, p = 0.01). All patients who were diagnosed with DGE or a grade B or C pancreatic leak but were discharged within goal LOS ≤ 5 were diagnosed upon their readmission. Pre-operative albumin < 3.5 g/dL was more frequent in those with LoS ≥6 days (19% vs. 44%, p = 0.01). Neither neoadjuvant chemotherapy (24% vs. 37%, p = 0.09), nor neoadjuvant radiation (20% vs. 20%, p = 1.0) was more common among those discharged in ≥ 6 days. There was no association between requiring a vascular resection and reaching goal LoS of ≤ 5 days or not (28% vs. 33%, p = 0.53). Higher scoring on ASA (p = 0.46) and ECOG (p = 0.36) were not associated with prolonged LoS.

Multivariate regression analysis was performed to evaluate factors that influenced LoS independent of the perioperative pathway. After controlling for the presence of diabetes, ASA class, use of neoadjuvant chemotherapy, and epidural use, patients treated on the PD pathway had three-times greater odds of having length of stay ≤5 days (Odds ratio (OR) 3.03, 95% Confidence Interval (CI): 1.41–6.53, p = 0.005). On univariate analysis, serum albumin level <3.5 g/dL was associated with longer length of stay and so when additionally controlling for hypoalbuminemia, the difference in length of stay was no longer significant (OR 2.07, 95% CI: 0.70–6.10, p = 0.19, likelihood ratio test p<0.001). However, because more than a third of patients in this study did not have albumin levels drawn peri-operatively, this likely represents introduction of type 2 error.

Risk factors for readmission at 30 days included blood transfusion in the OR (26.6% readmitted vs. 8.8% not, p = 0.009) as well as expected post-operative complications of any SAE, grade B or C pancreatic leak, DGE or need for TPN, and discharge to a place other than home (Table 3). Interestingly, ASA class was also a significant factor on univariate analysis, however there was a higher percentage of patients with ASA class 3 or greater in those who were not readmitted within 30 days. The significance of ASA class was not present for 90 day readmission, however, but both blood transfusion in the OR (25.0% readmitted vs. 9.0% not) as well as OR duration in minutes (519.6 vs. 438.9, p = 0.002) were significant (Table 4). Secondary outcomes of any SAE, grade B or C pancreatic leak, DGE or need for TPN, and discharge to a place other than home remained significant predictors of readmission at 90 days.

**Table 3:** Demographics, clinical characteristics, and outcomes of patients readmitted within 30 days of surgery.

	Readmitted within 30 days (n = 30)	Not readmitted within 30 days (n = 113)	p-value
**Demographics**			
Mean age, years (SD)	62.2 (9.6)	63.4 (10.6)	0.56
Female, n (%)	15 (50.0)	59 (52.2)	0.82
**Clinical characteristics**			
Mean BMI, kg/m2 (SD)	27.4 (5.2)	26.3 (5.5)	0.31
Mean smoking pack years (SD)	7.1 (12.0)	10.7 (21.9)	0.38
Diabetes, n (%)	6 (20.0)	24 (21.2)	0.88
Previous cardiac stent (%)	2 (6.6)	8 (7.0)	0.93
CKD, n (%)	0 (0.0)	4 (5.0)	0.35
Hypoalbuminemia <3.5 g/dL, n (%)	4 (26.6)	26 (34.2)	0.57
ECOG performance status, n (%)			0.88
0	10 (52.6)	38 (46.3)	
1–2	9 (47.3)	42 (51.2)	
≥3	0 (0.0)	2 (2.4)	
ASA class, n (%)			**0.02**
1	1 (3.3)	0 (0.0)	
2	8 (26.6)	28 (24.7)	
≥3	21 (70.0)	85 (75.2)	
Neoadjuvant chemotherapy, n (%)	9 (30.0)	35 (30.9)	0.91
Neoadjuvant radiation, n (%)	6 (20.0)	23 (20.3)	0.96
Preop epidural, n (%)	7 (23.3)	34 (30.0)	0.46
Mean OR duration, minutes (SD)	493.6 (159.8)	455.7 (127.6)	0.17
Mean IV fluid in OR, mL (SD)	5,697 (2,496)	5,255 (2,067)	0.32
Blood transfusion in OR, n (%)	8 (26.6)	10 (8.8)	**0.009**
Mean EBL in OR, mL (SD)	503.3 (761.6)	442.5 (771.2)	0.70
Soft pancreatic texture, n (%)	13 (44.8)	39 (39.7)	0.62
Pancreatic duct <3mm, n (%)	11 (37.9)	35 (33.6)	0.66
**Secondary outcomes**			
ICU admission POD1, n (%)	5 (16.6)	13 (11.5)	0.44
Serious adverse event, n (%)	23 (76.6)	21 (18.5)	**<0.001**
ISGPS 2016 grade B leak, n (%)	13 (44.8)	16 (14.2)	**<0.001**
ISGPS 2016 grade C leak, n (%)	6 (20.6)	6 (5.3)	**0.008**
Delayed gastric emptying grade B or C, n (%)	15 (51.7)	12 (10.7)	**<0.001**
Required TPN post-op, n (%)	13 (44.8)	11 (9.8)	**<0.001**
Discharge to other place than home, n (%)	5 (16.6)	3 (2.6)	**0.003**

PD, Pancreaticoduodenectomy; SD, Standard deviation; BMI, Body mass index; CKD, Chronic kidney disease; ECOG, Eastern Cooperative Oncology Group performance status; ASA, American Society of Anesthesiologists physical status classification; LOS, Length of stay; ISGPS, International Study Group of Pancreatic Surgery; IV, Intravenous; OR, Operating room; EBL, Estimated blood loss

**Table 4:** Demographics, clinical characteristics, and outcomes of patients readmitted within 90 days of surgery.

	Readmitted within 90 days (n = 40)	Not readmitted within 90 days (n = 88)	p-value
**Demographics**			
Mean age, years (SD)	62.4 (9.9)	63.8 (10.5)	0.47
Female, n (%)	20 (50.0)	44 (50.0)	1.0
**Clinical characteristics**			
Mean BMI, kg/m2 (SD)	27.0 (4.8)	25.9 (5.1)	0.25
Mean smoking pack years (SD)	6.2 (10.9)	12.5 (23.9)	0.11
Diabetes, n (%)	8 (20.0)	20 (22.7)	0.72
Previous cardiac stent (%)	3 (7.5)	7 (7.9)	0.92
CKD, n (%)	0 (0.0)	3 (4.8)	0.26
Hypoalbuminemia <3.5 g/dL, n (%)	6 (25.0)	23 (38.3)	0.24
ECOG performance status, n (%)			0.77
0	13 (48.1)	30 (45.4)	
1–2	14 (51.8)	34 (51.5)	
≥3	0 (0.0)	2 (3.0)	
ASA class, n (%)			0.23
1	1 (2.5)	0 (0.0)	
2	9 (22.5)	21 (23.8)	
≥3	30 (75.0)	67 (76.1)	
Neoadjuvant chemotherapy, n (%)	17 (42.5)	25 (28.4)	0.11
Neoadjuvant radiation, n (%)	11 (27.5)	16 (18.1)	0.23
Preop epidural, n (%)	11 (27.5)	28 (31.8)	0.62
Mean OR duration, minutes (SD)	519.6 (181.6)	438.9 (104.3)	**0.002**
Mean IV fluid in OR, mL (SD)	5,829 (2,700)	5,126 (1,864)	0.09
Blood transfusion in OR, n (%)	10 (25.0)	8 (9.0)	**0.01**
Mean EBL in OR, mL (SD)	676.2 (1,309)	384.4 (386.8)	0.06
Soft pancreatic texture, n (%)	22 (56.4)	47 (63.5)	0.46
Pancreatic duct <3mm, n (%)	14 (35.8)	27 (34.1)	0.85
**Secondary outcomes**			
ICU admission POD1, n (%)	7 (17.5)	11 (12.5)	0.45
Serious adverse event, n (%)	26 (65.0)	17 (19.3)	**<0.001**
ISGPS 2016 grade B leak, n (%)	13 (33.3)	14 (16.0)	**0.02**
ISGPS 2016 grade C leak, n (%)	7 (17.9)	5 (5.7)	**0.03**
Delayed gastric emptying grade B or C, n (%)	16 (41.0)	9 (10.3)	**<0.001**
Required TPN post-op, n (%)	14 (35.8)	8 (9,1)	**<0.001**
Discharge to other place than home, n (%)	5 (12.5)	2 (2.2)	**0.01**

PD, Pancreaticoduodenectomy; SD, Standard deviation; BMI, Body mass index; CKD, Chronic kidney disease; ECOG, Eastern Cooperative Oncology Group performance status; ASA, American Society of Anesthesiologists physical status classification; LOS, Length of stay; ISGPS, International Study Group of Pancreatic Surgery; IV, Intravenous; OR, Operating room; EBL, Estimated blood loss

## Discussion

This study demonstrates that our LoS of 5 days after pathway implementation is significantly shorter than our LoS prior to pathway initiation, as well as the NSQIP median of 8 days[5,23]. Equally as important, there was no significant increase in readmission rates at 30 and 90 days among those discharged by the goal of 5 days, indicating that patients were not discharged before medically ready. There was also no increase in serious adverse events, suggesting that the interventions involved in the perioperative pathway were safe. The reasons for our pathway's success is likely multi-factorial as previous studies with ERAS protocols have demonstrated, but the importance of pre-operative counseling of patients and their families as well as buy-in from clinical staff cannot be overstated.

In previous studies that have examined pancreatic surgery patients from 2005 to 2015, median LoS has varied widely from 7–27 days[5,23–26]. Most studies were similarly limited to being single institution teaching hospitals, with study size varying from 40 to 255 patients[5,26]. While some studies have demonstrated a small sub-group of patients (up to 9%) that perform exceptionally well after surgery and are discharged in 5 days or less, our study demonstrates that the majority (64%) of patients achieve this milestone in the setting of our peri-operative pathway[26]. Consistent with previous studies, we found no increase in readmission rates after earlier discharge[5,23,26].

Evaluating factors that were significantly related to LoS in previous studies, ASA class and ECOG score were not associated with attaining goal discharge in our patient population[27]. However, via multivariate analysis, pre-operative albumin level was found to be a significant predictor of meeting goal LoS. This correlation between lower albumin and longer LoS is similar to previous studies looking at albumin levels in patients with pancreatic cancer who underwent resection without neoadjuvant therapy, as well as patients undergoing colorectal and cardiac surgery[28–33]. Most studies defined hypoalbuminemia as serum level below 3.45 or 3.5 g/dL, although one used less than 3.0 g/dL[28–32]. Our data suggest that patients with serum albumin <3.5 g/dL prior to PD are less likely to make the goal of discharge by POD 5 and may benefit from additional nutritional counseling and possible supplementation prior to undergoing the stress of surgery. While we did not use pre-operative albumin at our institution to delay surgery nor do we have data on patients who delayed surgery, all patients during pre-operative counseling are encouraged to drink arginine-rich supplements prior to surgery and focus on protein-rich nutrition. Some barriers to patients actually receiving the shakes included availability at pharmacies and insurance coverage. We do not have data on who was unable to obtain the specific shakes, and therefore approached this with an "intention to treat" mindset, but the emphasis on adequate protein and calorie nutrition before and after surgery was achieved regardless. Additionally, patients were encouraged to meet the criteria of walking 2 miles before surgery. Also in agreement with most previous studies, discharge within goal of 5 days was not influenced by neoadjuvant systemic chemotherapy or radiation[26,33–36].

Regarding intra-operative and post-operative factors, we did not see a difference in morbidity, readmission, or meeting discharge goal of 5 days in patients who underwent vascular reconstructions compared to those who did not unlike previous studies[14,37]. The placement and duration of NG tubes is widely discussed in many fast-track pathway with the goal of early post-operative feeding that has been shown in numerous studies to shorten the time to return of bowel function[10,15,38–40]. Intra-operative NG tube placement did decrease following pathway implementation although they were still placed in some cases by surgeon judgment. Additionally, NG tubes were placed post-operatively if indicated in the setting of prolonged ileus or delayed gastric emptying as appropriate, but these rates did not differ pre- and post-pathway which, along with unchanged pancreatic leak rate, suggests no significant changes in surgical techniques were made during pathway implementation. Fluid overload has also been shown to increase morbidity and LoS after PD due to pulmonary complications and increased ileus as mentioned above, and thus the increase in early enteral feeding and decrease in intravenous fluids was strictly regulated in our pathway[17,41,42]. Avoidance of TPN if at all possible contributes to meeting fluid goals and decreasing infectious risks, and there was no difference in initiating TPN after pathway implementation.

Interestingly, while epidural use is promoted in many colorectal pre-operative pathways, including at our institution, their use for PD patients has been decreasing since early 2015. This preference against epidurals has grown without explicit direction from the perioperative pathway stakeholders, however it may influence our results associating epidurals with increased risk of not discharging by POD 5. In contrast, a previously reported single institution study examining Whipple patients with LoS ≤ 5 days found a shorter LoS was associated with higher rates of epidural placement[25]. Additionally, other studies examining pain control and respiratory complications after pancreaticoduodenectomy have shown benefits to epidural placement, although some did note slightly increased LoS[15,43–45]. However, it has been shown that ineffective epidurals often lead to decreased pain control and therefore decreased patient mobility post-operatively[44–46]. Additionally, hypotension associated with epidural use can lead to increased IV fluid administration and increased LoS[46]. It is possible that interventions to correct hypotension or only partial pain control with some epidurals led to a longer LOS in our study. Successful pain control and avoidance of complications with epidural analgesia is likely to be highly dependent on institutional experience with catheter placement.

All of our patients had a drain placed near the site of the pancreaticojejunostomy intra-operatively. Drain fluid amylase was routinely checked prior to pathway initiation, but this was standardized to POD 1 and POD 3 with the pathway. Drains were removed on POD 3–4 if the fluid amylase was <3 times the higher of the serum amylase or the upper limit of normal and there was no concern for leak based on appearance of the output. Using the ISGPS definition of pancreatic leak, which has been found to predict LoS > 10 days in previous studies, we did find a significant association with grade B or C leak and LoS within goal of 5 days or readmission; many of these leaks were diagnosed upon readmission regardless of initial LOS, however[21,47,48]. Patients with higher drain amylases on POD 3 may not have been discharged as readily for fear of development of a leak or need for additional teaching to go home with a drain, but there is the possibility that the drain was not appropriately functioning and readings could have been misleadingly low.

There are several limitations to our retrospective study. While we officially adopted the perioperative pathway on June 1st, 2015, it is possible that culture change occurred prior to actual pathway initiation that may have influenced the study results. Additionally, epidural placement strongly declined during the months prior to pathway implementation. While this was not part of the pathway design, it inadvertently changed patient management outside of the pathway that had an effect in univariate analysis.

## Conclusions

The coordination of our peri-operative care system helps set patient expectations and optimize care after pancreaticoduodenectomy. With this investment from all members of the care team, LoS has been lowered to a median of 5 days without compromising safety. Further improvements may be achieved by aggressively optimizing nutrition prior to surgery.

## Supporting information

S1 Fig Care map for patients. (PDF)
