# Supplementary material for: Standardization of perioperative care facilitates safe discharge by postoperative day five after pancreaticoduodenectomy
Source: PLoS One. 2018 Dec 28;13(12):e0209608. doi: 10.1371/journal.pone.0209608 (PMC6310358; doi:10.1371/journal.pone.0209608)
Supplement: S1 Fig — (PDF) [file pone.0209608.s001.pdf]

UW Medicine
UNIVERSITY OF WASHINGTON MEDICAL CENTER

Pancreaticoduodenectomy/Whipple CareMap: Your Hospital Stay

How to prepare and what to expect

Before Surgery Day

- ❑ We will schedule your follow-up visit for after surgery – this date may change, depending on how long you are in the hospital.
- ❑ Quit smoking at least 2 to 4 weeks before surgery.

Starting 7 to 14 days before surgery:

- ❑ Walk 2 miles or at least 20 minutes a day.

7 days before surgery:

- ❑ Stop taking aspirin and NSAIDS (*non-steroidal anti-inflammatory drugs*) such as ibuprofen (Advil, Motrin) and naproxen (Aleve, Naprosyn)

Starting 5 days before surgery:

- ❑ Drink your Impact Advanced Recovery drink 3 times a day, as well as your regular meals.

Starting 2 days before surgery:

- ❑ Do **not** shave near the surgical areas.

Day before surgery:

- ❑ Receive a call from the hospital with your assigned arrival time.
- ❑ **Before you go to bed**, take a shower with the chlorhexidine gluconate (CHG) soap:
  - Shower and shampoo with your regular soap
  - Rinse well
  - Wet a clean washcloth, then turn the shower off
  - Pour 1/2 bottle of CHG on the washcloth and use the washcloth to wash from your shoulders to your knees – include your groin crease, but **not** your private parts
  - Leave the soap on your skin for 1 minute
  - Rinse well
- ❑ **Before midnight**, drink one 8-ounce bottle of apple juice. After midnight, you may have only clear liquids, nothing else by mouth.

Surgery Day

Before you leave home:

- ❑ Take another shower using the same steps as last night.
- ❑ Do **not** apply deodorant, lotions, scents, or hair products after your shower.

Starting 2 hours before your surgery, do not eat or drink anything EXCEPT:

- ❑ Right after you park at the hospital, drink one 8-ounce bottle of apple juice.

At the hospital:

- ❑ Check in at Surgery Registration (Surgery Pavilion, 2nd floor) at your assigned arrival time.
- ❑ An *intravenous* (IV) line will be placed in your arm to give you antibiotics and fluids.
- ❑ We will give you a heating blanket to keep you warm, improve healing, and lower infection risk.
- ❑ Your surgeons and other members of your care team will meet with you to answer any questions you have.
- ❑ The Anesthesia team will talk with you about the *anesthesia* (sleeping medicine) you will receive during surgery.
- ❑ The Anesthesiology team will take you to the operating room.
- ❑ We will take your family or friends to the surgery waiting room.

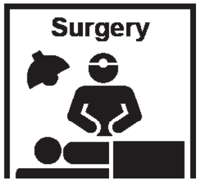

During surgery, your doctors will inject Exparel (a numbing medicine) along your incision. This will help control pain for 48 to 72 hours after surgery.

After surgery, you will:

- ❑ Wake up in the recovery room, where you will stay for several hours
- ❑ Move to your hospital room when you are awake and comfortable, and your vital signs are stable

You will have:

- ❑ An IV in your arm to give you fluids and medicines
- ❑ A *patient-controlled analgesia* (PCA) pump so that you can give yourself pain medicine through your IV as needed
- ❑ Wraps on your legs while you are in bed – these wraps fill with air to help with blood flow and lower the risk of blood clots
- ❑ A *Foley catheter* (tube) in your bladder to drain urine (this was placed during surgery)

Your nurse will:

- ❑ Help you sit up on the edge of your bed
- ❑ Remind you to take sips of clear liquids and chew on ice chips and gum to get your digestion working
- ❑ Teach you how to use an incentive spirometer (blue breathing device)

<table>
<tr><th></th><th>Day 1</th><th>Day 2</th><th>Days 3 and 4</th><th>Discharge Day</th><th>At Home</th></tr>
<tr><td>Medicines/Treatments</td><td colspan="2">❑ You will have control of giving yourself pain medicine as needed through your PCA pain pump.
❑ Foley bladder catheter will be in place. It will be removed by Day 2.</td><td>❑ When you can handle solid food, PCA will stop and you will take pain pills by mouth.
❑ A pharmacist will review Lovenox (blood thinner) information.
❑ Your nurse will teach you how to give yourself Lovenox injections.</td><td>Discharge will be Day 4 or 5, based on progress.
❑ Your doctor will prescribe a stool softener.
❑ Receive prescription pain medicine (opioids).
❑ Receive Lovenox medicine to last 28 days.</td><td rowspan="5">Self-care
❑ Give yourself 1 shot of Lovenox every day for 28 days.
❑ Take a stool softener while taking opioids. Stop taking stool softener if you have diarrhea.
❑ If you are constipated (hard stool or bowels will not empty), try senna, Miralax, or Milk of Magnesia.
❑ Start to taper opioids. Take only as needed.
❑ Take ondansetron (Zofran) for nausea.
❑ Take pantoprazole every day unless instructed otherwise.
❑ Eat 5 to 6 small meals a day.
❑ Walk as much as you can.
Cautions
❑ For 6 weeks: Do not lift anything that weighs more than 10 pounds (1 gallon of milk weighs almost 9 pounds).
❑ Do not drink or drive while taking opioids.
Questions or Concerns
❑ Dr. Park's patients: Call 206.598.4477
❑ Dr. Pillarisetty's patients: Call 206.606.7555
Follow Up
❑ Go to follow-up clinic visit 1 to 2 weeks after discharge.</td></tr>
<tr><td>Diet</td><td>❑ Start clear liquid diet. Do not drink more than 8 ounces in 8 hours (about 30 mL an hour).</td><td colspan="2">❑ Talk with nutritionist about food choices, portions, and how often to eat.
❑ When handling liquids, move to regular diet.</td><td>❑ Keep eating a regular diet. Avoid concentrated sugars.</td></tr>
<tr><td rowspan="2">Activities and Self-care</td><td colspan="3">❑ Use your spirometer 10 times every hour to keep fluid out of your lungs.
❑ Do not get out of bed without a nurse's help.
❑ Your goal is to be out of bed for a total of 6 hours a day. We will help you:
- Sit up in a chair for all meals.
- Take 3 to 4 walks a day.
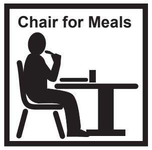

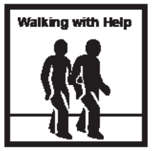

❑ Sponge bath.
❑ Daily weighing.
❑ Receive diabetes education for diet and insulin on Day 3 (if needed).</td><td rowspan="2">❑ Shower and dress in your own clothes by about 9 a.m.
Discharge goals are met:
❑ Handling your diet
❑ Pain under control
❑ Getting around OK
❑ Passing gas, having bowel movements
❑ Received diabetes and pharmacy teaching (if needed)
❑ Follow-up clinic visit set for 1 to 2 weeks after discharge</td></tr>
<tr><td colspan="3">Day 1: ❑ Aim to walk ½ mile.
❑ An Occupational Therapist (OT) will evaluate you and set up a plan.
❑ A Physical Therapist (PT) will evaluate you and set up a plan.
Days 2, 3, 4: ❑ Aim to walk 1 to 1½ miles each day.
Day 4: ❑ Shower with help.</td></tr>
<tr><td>Planning</td><td colspan="2">❑ Know your discharge goals:
- Handling regular foods
- Passing gas and having bowel movements
- Pain under control
- Walking by yourself</td><td>❑ Meet with social worker to talk about home healthcare or skilled nursing facility (if needed).</td><td>❑ Plan for your ride home from the hospital.</td></tr>
</table>

© University of Washington Medical Center
Published PFES: 05/2013, 05/2015, 07/2016, 10/2016, 03/2017, 05/2017, 01/2018, 08/2018
Clinician Review: 08/2018
Reprints on Health Online: https://healthonline.washington.edu
